# Scalable Fire and Smoke Segmentation from Aerial Images Using Convolutional Neural Networks and Quad-Tree Search

**DOI:** 10.3390/s22051701

**Published:** 2022-02-22

**Authors:** Gonçalo Perrolas, Milad Niknejad, Ricardo Ribeiro, Alexandre Bernardino

**Affiliations:** Instituto de Sistemas e Robótica, Instituto Superior Tecnico, University of Lisbon, 1049-001 Lisbon, Portugal; goncalo.perrolas@tecnico.ulisboa.pt (G.P.); ribeiro@isr.tecnico.ulisboa.pt (R.R.); alex@isr.tecnico.ulisboa.pt (A.B.)

**Keywords:** fire detection, smoke detection, aerial images, wildfire, convolutional neural networks

## Abstract

Autonomous systems can help firefighting operations by detecting and locating the fire spot from surveillance images and videos. Similar to many other areas of computer vision, Convolutional Neural Networks (CNNs) have achieved state-of-the-art results for fire and smoke detection and segmentation. In practice, input images to a CNN are usually downsized to fit into the network to avoid computational complexities and restricted memory problems. Although in many applications downsizing is not an issue, in the early phases of fire ignitions downsizing may eliminate the fire regions since the incident regions are small. In this paper, we propose a novel method to segment fire and smoke regions in high resolution images based on a multi-resolution iterative quad-tree search algorithm , which manages the application of classification and segmentation CNNs to focus the attention on informative parts of the image. The proposed method is more computationally efficient compared to processing the whole high resolution input, and contains parameters that can be tuned based on the needed scale precision. The results show that the proposed method is capable of detecting and segmenting fire and smoke with higher accuracy and is useful for segmenting small regions of incident in high resolution aerial images in a computationally efficient way.

## 1. Introduction

Over the last two decades, automated systems to support forest fire fighting have shown an increasing popularity as a research topic due to the growing incidence of forest fires around the world. One example operating in Portugal is the project CICLOPE [1] that monitors 1,300,000 hectares of forest and detects the occurrence of fire through smoke analysis on the images captured. Recently, Unmanned Aerial Vehicles (UAVs) equipped with imaging sensors have become popular in diverse forest and agriculture applications [2,3], including the detection and monitoring of wildfires [4] Fire and smoke detection methods in images are the essential part in these systems, which are based on Artificial Intelligence (AI).

The classical methods for fire detection were mainly based on handcrafted features obtained based on RGB color values [5,6,7]. The features are then classified using vector classifier methods such as Support Vector Machines (SVM). Recently, Convolutional Neural Networks (CNNs) have led to state-of-the-art results for fire and smoke recognition [8,9], similar to many other computer vision areas. Beyond recognition, it is also very important for an autonomous system to determine the location of the fire or smoke. There are some works which proposed to localize fire by bounding box [10] or pixel segmentation [11]. For fire segmentation, the approaches are mainly based on adapting multi-label segmentation methods such as deeplab-v3 [12] for the case of fire or smoke segmentation [11].

The majority of fire/smoke detection systems rely on images captured by fixed cameras at high altitude in order to cover large areas of forest terrain, resulting in high resolution surveillance images.

Although theoretically the input size of the images in CNNs could be arbitrary for a learned network, in practice, the images are usually downsized to small sizes (e.g., 244×244 pixels in VGG [13]) to feed into CNNs. Using high resolution images causes a large increase in the computational time, especially in training phases to compute the gradient of the weights. It also highly increases the memory usage, which is another limitation. Although in many applications it suffices to have small image sizes, for the applications demanding high resolution aerial images to detect fire and smoke, image resizing may result in missing small fire areas, and decreases the georeferencing accuracy. Detecting small areas of fire is important to detect the fire at the early phases of an incident and timely activate alarms. On the contrary, if the smoke/fire area is noticeable, resizing the image to a smaller size may lose accuracy of the localization, especially in the boundaries of smoke or fire. Existing methods to localize fire do not address the ability to detect variable sizes of fire in images, which we denote as “scalability”.

In this paper, we propose a novel scalable method for the detection and segmentation of smoke and fire in images based on the well-known quad-tree [14] search algorithm. The proposed network consists of two classification and segmentation CNNs to detect and localize fire and smoke. The method processes image patches, and begins by considering the entire image as a patch. For each patch, the classification network first determines whether the patch contains instances of fire or smoke. If fire/smoke is detected, the segmentation network computes the initial segmentation of fire. If the the segmented area in the patch is small (below some threshold), the image is sliced into four parts, and each part is “zoomed-in” to search for smaller scale instances of interest. The patches are finally aggregated to form the entire segmented mask for the image. Note that, in all mentioned stages, the inputs of CNN are fixed-size images that are downsized versions of the selected patches to keep the computational efficiency. The results show that the proposed method is capable of segmenting fire and smoke in images with high accuracy, and is able to localize very small areas of fire or smoke in images, which is very important for early detection systems.

## 2. Related Works

Classical methods for fire and smoke detection mainly use image processing techniques in which the images are analyzed by texture, contrast, or RGB components of pixels to detect fire and smoke [5,6,7,15]. The features extracted from the color of pixels, such as color histograms, can be used to distinguish fire regions from the background. So, many methods use features calculated from the RGB components of pixels to detect fire [5,16] or smoke [17].

Recently, state-of-the-art methods for fire detection, similar to many other areas of computer vision, are based on CNNs [18]. These methods do not require hand-crafted features, as the features are learned automatically by an end-to-end supervised training scheme. The method in [8] uses simplified structures of the Alexnet [19] and Inception networks [20] for classifying images to fire and non-fire. In [10], the faster Region-CNN (R-CNN) is first used to extract fire candidate regions, and then each region is classified. Some methods consider the classification of fire videos using CNNs [21,22]. In [22], a long short term memory network (LSTM) is used for fire classification.

Apart from classification, some methods consider the problem of segmentation of fire or smoke regions in images, as it enables the system to localize the incident regions. This localization can be used for georeferencing the events from aerial images. Deeplabv3+ network and Squeeze-net have been employed in [11,23], respectively, to segment the fire regions. Similar methods based on CNNs have also been proposed for smoke segmentation. Reference [24] used two segmentation networks to infer a segmentation mask based on coarse and fine features extracted from smoke images, and then fused them using a new network.

Quad-Tree is a well-known data structure for storing and retrieving two dimensional data [14]. It partitions two-dimensional data recursively into four quadrants or regions and unify ’interesting’ data into one cell. It has lots of applications in different areas such as image compression [25] and data storage [26]. One interesting feature of Quad-tree in our application for fire/smoke detection is the ability of efficient search of specific regions in images.

## 3. Methodology

The proposed method is designed to process images taken from an aerial point of view, either taken from a static surveillance tower, or a conventional aerial vehicle like an airplane or helicopter or an unmanned vehicle like a drone. Those vehicles must be equipped with an RGB camera with enough resolution to be able to detect small fire/smoke areas and a transmission system to send the images to a processing unit on land. The aerial images will then go through the proposed algorithm to detect fire and smoke.

Although in theory the convolutional filters obtained by CNNs can be used for any input size, in practice, the images are downsized to lower resolutions, e.g., 244×244 to feed into the CNNs. Considering high resolution images for the input of a CNN causes high computational complexity for training and inference. Moreover, convolutional filters obtained from images with one specific scale of an object may not perform optimally for detecting other scales of the object. Very recently, some methods have considered this problem and aim to extract information from high resolution images using CNNs [27,28]. However, these methods are proposed for classification and not for segmenting the objects. The size of the areas of fire and smoke in aerial images may span several orders of magnitude, so it is important to detect small regions of fire/smoke from high resolution images. Those areas may even disappear in the first step of downsizing the image or become very difficult to detect.

The main purpose of this algorithm is to efficiently segment small areas of fire and smoke in high resolution images. A straightforward approach would be to slice (divide) the image into patches of fixed-size, then feed each slice as an input to the CNN, and finally aggregate the output patches by returning them to the original position of the patch in the image. However, this approach is not adaptive to the scale of the events, and is time consuming in the test phase. In this paper we propose to use a search method based on the Quad-tree approach. Depending on the size of the fire/smoke area in the image, the algorithm will tend to either do a more precise detection, involving zoomed-in patches, or a more global search using patches from larger areas or even the whole image.

The proposed method, described in Algorithm 1, uses two CNNs in the process, one for classification and one for segmentation, which are trained independently. Starting from the entire image as a patch, the basic operation of the proposed method is composed of three steps. The first step classifies the input patch to check if fire/smoke. If fire/smoke is detected, then the second step uses the segmentation network to compute a binary mask marking where fire/smoke exists inside the patch. In the third step, if the area of the segmented mask is lower than a minimum segmentation threshold, meaning that a more detailed analysis of the event is required, the patch is sliced into four parts and the procedure is repeated for the four patches. In all stages the patch is downsized to the input size of classification and segmentation networks.

In basic procedure of the algorithm, the classification network decides if the fire exists in the patch in order to move to the next stage for the segmentation. If patches are too large, small areas of fire may be lost by the classification network, resulting in false negative classifications. To resolve this issue, we propose to use a maximum size for the patches to be classified as negative, above which we further divide the patches to higher scales even if the output of the classification network is negative. This distinguishes patch sizes that have high false negative rates, and therefore should be further analyzed, from patch sizes that have low false negative rates, and can be confidently discarded when classified as negatives. To control the maximum resolution of analysis, we also define a minimum patch size threshold for the networks, denoting when the algorithm stops further dividing the patches. This threshold determines the precision of the algorithm, and could be tuned based on the precision required for the application at hand.

**Figure 1:**
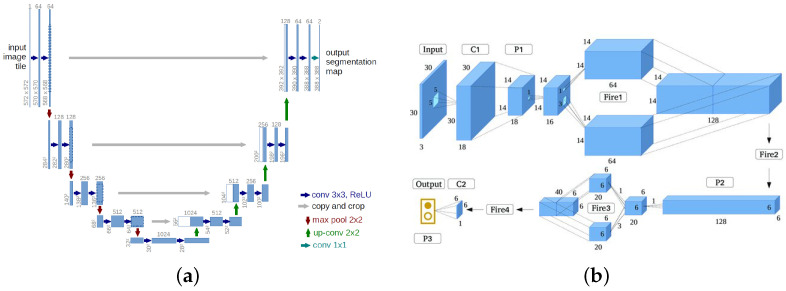
Network structures of segmentation and classification network used in our method. (**a**) Segmentation network [30], (**b**) Classification Network SqueezeNet [29].

**Figure 2:**
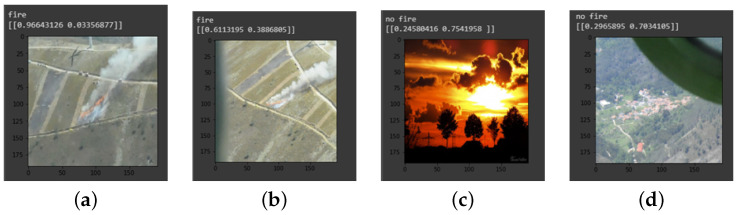
Examples of classifications using SqueezeNet in our method. (**a**) Fire image (Positive), (**b**) Fire image (Positive), (**c**) Red sky (Negative), (**d**) Red rooftops (Negative).

For classification, SqueezeNet [29] is used due to high computational efficiency and acceptable performance in our application. U-net [30] is used for segmentation. Figure 1 shows the structure of the networks used for segmentation and classification in our method. Both networks are trained independently using fire and smoke image datasets, suitable for each specific task. Figure 2 shows some examples of the output of SqueezeNet.
**Algorithm 1:** Quad_Tree Algorithm.
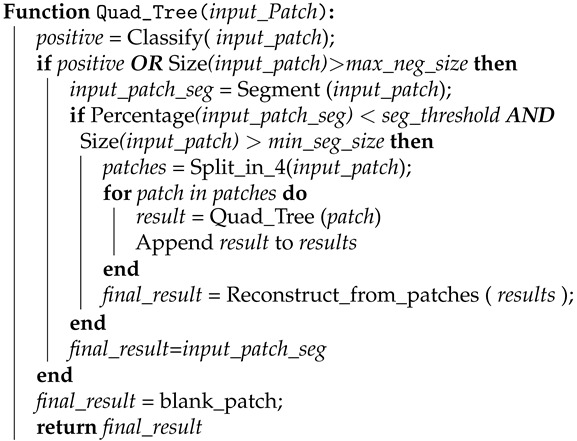


Below we describe the functions and parameters used in Algorithm 1.

- Percentage() computes the ratio between the number of segmented pixels divided by the total number of pixels in the patch;
- seg_threshold is the threshold value for the previous ratio, below which the algorithm continues the ’zooming’ process;
- max_neg_size is the maximum value of the patch sizes for which we rely on the negative classification output;
- min_seg_size is the minimum value of patch size for the zoom-in process. This is actually the minimum size of patch the algorithm can reach.

The last three mentioned parameters can be tuned based on the application at hand, and could be defined to set the trade off between the computational complexity and the accuracy of the algorithm.

## 4. Implementation and Results

### 4.1. Dataset

In order to train and evaluate the networks, we created a labeled dataset of fire and smoke images, including challenging negative images. For the classification network, the labels indicate if the image contains the phenomenon or not. For the segmentation networks, the labels specify each pixel class. In total, four different types of datasets were gathered for the tasks of fire classification, fire segmentation, smoke classification, and smoke segmentation.

The images gathered for the fire segmentation dataset mainly came from two sources: Corsican dataset [31] (RGB images with pixel wise labelling), and a batch of images gathered online that were manually labeled to extend the size of the dataset. For the smoke dataset, we used three sources: the datasets of [32,33] augmented by 300 images gathered online and segmented manually. The labeling was done using the Image Labeler App included in the Computer Vision System Toolbox 8.0 from Matlab [34]. We have included challenging negative examples in the dataset, which are likely to produce false positive results. In case of fire, we have included images containing sunsets/sunrises, reddish color sky, red foliage, and red objects that can appear in fire fighting situations (such as fire trucks, airplanes, and helicopters). For smoke, the images that are likely to produce false positive results are clouds. By including them in the dataset, the CNNs are trained to distinguish those images from real fire and smoke examples. Two examples are shown in Figure 2, which the classification network is able to classify correctly. We have also included images that add diversity of situations not covered in the Corsican Dataset, mainly images taken from long distances, from an aerial perspective, and with small areas of fire/smoke. In Table 1, an overview of our datasets is presented, including the number of images gathered for each category. In our experiments, the dataset is divided randomly into train/validation/test subsets with 70, 20, and 10 percentage of images, respectively.

### 4.2. Networks Training

We have implemented the code in Python with the Tensorflow library, running on a single core hyper threaded Xeon Processor at @2.3Ghz and a Tesla K80 (GPU). As explained earlier, the algorithm uses in total four different network instances, each one having a different task. Two instances of the SqueezeNet and two instances of the U-Net were trained. The input size for the segmentation network is 128×128, and for the classification network is 192×192. We chose a higher input size for the classification network as it assumes a key role in the pipeline by making the decision of whether the algorithm should proceed to the segmentation step. To train the networks to adapt to different scales of instances of fire and smoke, we sample random patches of different sizes from the training images, and resize them to the input size of the network.

The training parameters for the classification networks were set as follows,

**Fire Classifier Training Parameters:**

- **Optimizer**: Adam()
  Learn. Rate: 0.001
  $\beta_{1}$: 0.9;
  $\beta_{2}$: 0.999;
  ϵ: 1e-7;
- **Loss**: Binary Crossentropy;
- **Batch Size**: 32;
- **Patience**: 20;
- **Epochs**: 150;
- **Monitor**: Val. Loss;

**Smoke Classifier Training Parameters:**

- **Optimizer**: Adam()
  Learn. Rate: 0.002
  $\beta_{1}$: 0.9;
  $\beta_{2}$: 0.999;
  ϵ: 1e-7;
- **Loss**: Binary Crossentropy;
- **Batch Size**: 32;
- **Patience**: 20;
- **Epochs**: 150;
- **Monitor**: Val. Loss;

The parameters used in the training phase for the U-net segmentation network are as follows,

**Fire Segmentation Training Parameters:**

- **Optimizer**: Adam()
  Learn. Rate: 0.0001
  $\beta_{1}$: 0.9;
  $\beta_{2}$: 0.999;
  ϵ: 1e-5;
- **Loss**: Binary Crossentropy;
- **Batch Size**: 32;
- **Patience**: 30;
- **Epochs**: 200;
- **Monitor**: Val. Loss;

**Smoke Segmentation Training Parameters:**

- **Optimizer**: Adam()
  Learn. Rate: 0.0005
  $\beta_{1}$: 0.9;
  $\beta_{2}$: 0.999;
  ϵ: 1e-5;
- **Loss**: Binary Crossentropy;
- **Batch Size**: 32;
- **Patience**: 50;
- **Epochs**: 200;
- **Monitor**: Val. Loss;

The parameters were chosen to lead to an acceptable performance on the validation set. The training took approximately two and a half hours for fire and nearly two hours for smoke.

In order to measure the performance of the classification network, which has an important role in our algorithm, the accuracy of the classification network in the training validation and tests set are shown in Table 2. The confusion matrix for the test set is also shown in Table 3 and Table 4. Note that in this experiment all images are downsized to the classification network input size.

### 4.3. Results

In this part, we evaluate the overall performance of the proposed system and its performance when some of its components are disabled. We use the datasets reserved for testing proposes. The evaluation of each class is made separately and, for each, we tested four different models:

- **1. Q + C + S**: This model corresponds to the complete proposed algorithm. It uses all the components explained earlier, the QuadTree algorithm, the classification stage, and the segmentation stage;
- **2. Q + S**: This model removes the classification component from the algorithm, and QuadTree assumes the classification to always be positive;
- **3. C + S**: In this model, QuadTree methodology is removed. It divides the image into patches of minimum size (min_seg_size). It then processes all patches by first classifying them and then segmenting them.
- **4. S**: This final model consists only of the segmentation stage with the input image resized to the network input size. This corresponds to conventional semantic segmentation methods (in our case with Deeplab-v3).

In the experiments, the seg_threshold parameter is 30%, max_neg_size, and min_seg_size are set to 512×512, and 128×128, respectively (see Table 5).

To evaluate the performance of the four different implementations we use the mean and standard deviation (SD) of the Intersection over Union (IoU) metric together with the pixel accuracy and processing time per pixel. IoU is the common metric used to measure the quality of segmentation methods through the amount of overlap between the computed segmentation and the ground truth mask. It is computed by the ratio of the intersection over the union of the segmentation area with respect to the ground-truth mask area. The mean IoU is the mean value of the IoU and SD is the standard deviation of the IoU, computed across all images of the test set. The higher the mean IoU, the better the average performance of the method is. The higher the SD, the bigger the variability of the quality of the segmentation from frame to frame. To compute IOU, all obtained masks are resized to the size of the original image (mask). In order to compare the computational complexity, the average processing time per pixel is computed, as the resolution of processed images are different in each implementation.

The results are reported in Table 6 and Table 7, for fire and smoke, respectively. As mentioned earlier, S in the table corresponds to normal segmentation methods. Here we used Deeplab-v3 [12]. C + S corresponds the the exhaustive search by processing all patches with the minimum size.

As it can be seen in Table 6 and Table 7, the complete system has a better performance in terms of IOU compared to the other models for the smoke. For fire segmentation, the C + S (exhaustive search) method performs slightly better. However, using the quad-tree significantly improves the speed (more than three times in both cases). The simple segmentation in our results has less computational complexity, as it only requires one pass to the network for evaluation. However, using the Quad-Tree leads to a considerable improvement over the segmentation-only method (S) for fire (improvement from 83.49 to 88.3 in IoU). Moreover, in many cases the segmentation network cannot detect small portions of fire/smoke incidents from aerial images, which is a great limitation for early detection of wildfires. Similarly, for smoke segmentation, although the time complexity is slightly higher, the performance has improved from 77.21 to 83.37 in IoU compared to conventional Deeplab-v3 segmentation.

**Figure 3:**
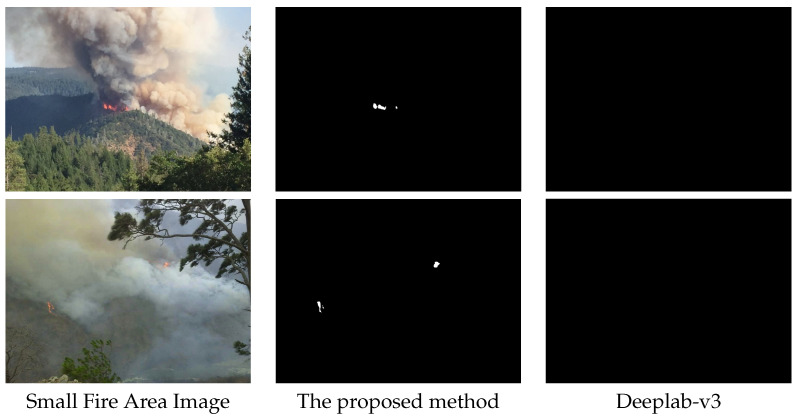
Examples of segmentation of small regions of fire in images covering large areas of terrain.

Figure 3 shows the performance of the proposed method for images containing small fire parts compared to DeepLab-v3 [12], which is a well-known and convensional image segmentation method. As it can be seen, the proposed method is capable of segmenting the fire part of the image while Deeplab fails to do so.

**Figure 4:**
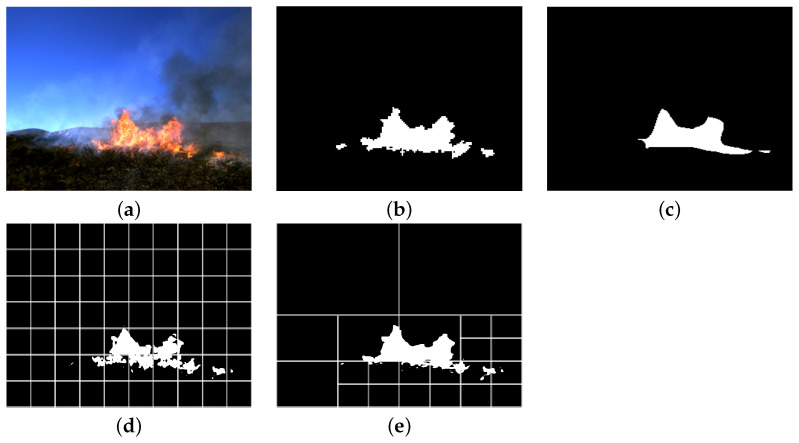
Comparison of the segmented masks produced by different methods and visualization of the searches. The searched regions are indicated by white borders. (**a**) Input Image, (**b**) Ground-truth mask, (**c**) DeepLab v3 [12], (**d**) Exhaustive search (C + S), (**e**) Proposed quad-tree search (Q + C + S).

Figure 4 shows illustrated segmentations obtained by the proposed method compared to DeepLab-v3 [12]. Patches used by the quad-tree algorithm are indicated with white border lines. As it can be seen, image regions with smaller fire areas are more refined by the proposed algorithm, leading to more precise segmentation. The parts in the image where there is no fire or where there are larger areas of fire are more coarsely segmented. It can be seen that the proposed method has better precision, especially in the boundaries of fire compared to DeepLab-v3.

**Figure 5:**
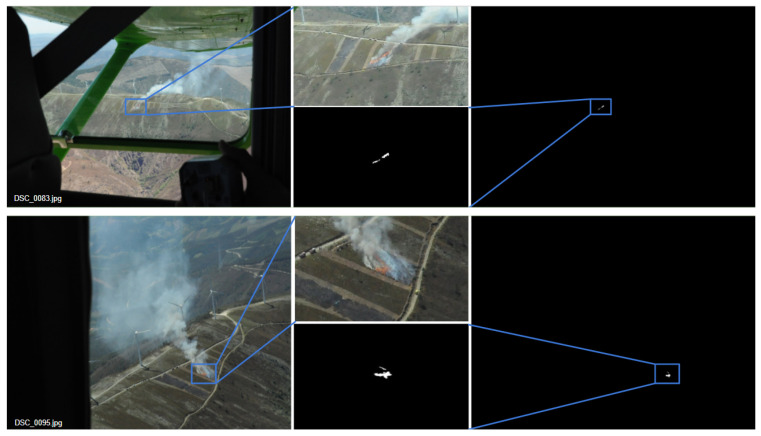
Two examples of fire segmentation of the proposed method for aerial images containing a small portion of fire. (**Left**): Original images; (**Right**): Segmented masks by Q + C + S; (**Middle**): Magnified images and obtained masks.

Figure 5 shows two examples in which the fire areas are very small (to better visualize the results, we added a zoom crop). In these examples, it can be seen that the proposed method is capable of segmenting the incident parts with high level of details.

**Figure 6:**
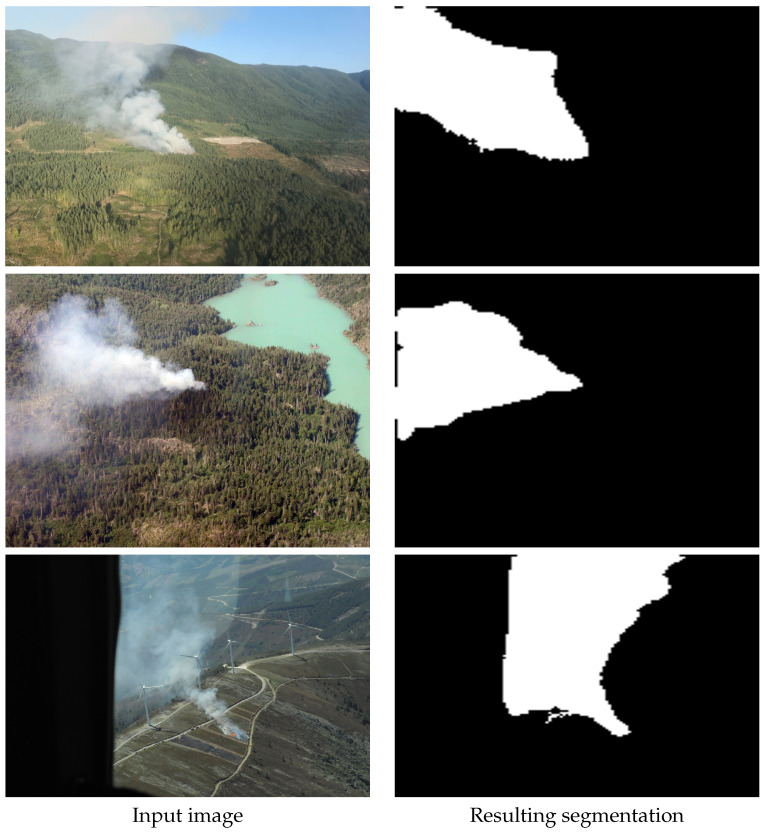
Example of segmentation of smoke by the proposed method.

**Figure 7:**
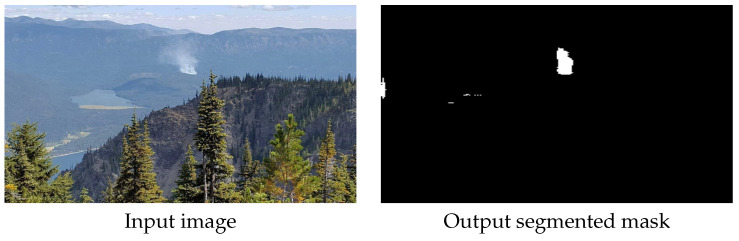
Example of segmentation of small smoke area by the proposed method.

Smoke segmentation examples in images captured from an aerial point of view are shown in Figure 6. Here we show some examples of smoke segmentation performed by our proposed method. These images are captured by many common surveillance systems such as fixed mounted cameras on hill tops or gimbal systems installed in manned and unmanned aerial vehicles. An example of segmentation of images containing small portion of smoke is shown in Figure 7.

## 5. Conclusions

We proposed in this paper a computationally efficient method based on quad-tree search to localize and segment fire and smoke on different scales. Unlike exhaustive search, quad-tree searches for the incidents work adaptively on different scales, leading to a computationally efficient algorithm. The results show that the algorithm is capable of detecting fire and smoke regions in aerial images in cases when the fire and smoke regions are small compared to the image size. This problem, to the best of our knowledge, has not been addressed in the literature, but is relevant to the efficient and early detection of wildfires. Using the proposed method for other types of images such as infra-red images could be a subject of future works.

## Figures and Tables

**Table 1:** The number of images gathered in the dataset in each category.

Classification	Fire	Positive	800
		Negative	520
	Smoke	Positive	500
		Negative	300
Segmentation	Fire	Containing fire	700
		Negative	450
	Smoke	Containing fire	300
		Negative	60

**Table 2:** The performance of the SqueezeNet classification network in our dataset.

	Fire Dataset	Smoke Dataset
Training set accuracy	98.56	96.10
Validation set accuracy	95.98	91.01

**Table 3:** Normalised confusion matrix of the test set for the fire dataset.

	Predicted Fire	Predicted Non-Fire
True fire class	0.951	0.049
True non-fire class	0.041	0.959

**Table 4:** Normalised confusion matrix of the test set for the smoke dataset.

	Predicted Smoke	Predicted Non-Smoke
True smoke class	0.902	0.098
True non-smoke class	0.087	0.913

**Table 5:** The parameters used in the algorithm producing the reported results.

seg_threshold	max_neg_size	min_seg_size
30%	512×512	128×128

**Table 6:** The effect of adding different stages to the proposed method and comparison to common segmentation (fire dataset).

Model	Mean IoU %	SD (of IoU)	Pixel Acc.	Process. Time (per Pixel)
Q + C + S	88.3	**0.10**	95.8	$5.4\times 10^{-6}$
Q + S	88.01	0.15	95.8	$6.0\times 10^{-6}$
C + S	**88.51**	**0.10**	**95.9**	$18.4\times 10^{-6}$
S	83.49	0.22	91.3	$4.2\times 10^{-6}$

**Table 7:** The effect of adding different stages to the proposed method and comparison to common segmentation (Smoke dataset).

Model	Mean IoU %	SD (of IoU)	Pixel Acc.	Process. Time (per Pixel)
Q + C + S	**83.37**	**0.133**	**91.6**	$5.3\times 10^{-6}$
Q + S	82.81	0.149	91.5	$6.1\times 10^{-6}$
C + S	83.25	0.144	91.6	$18.3\times 10^{-6}$
S	77.21	0.215	87.4	$4.2\times 10^{-6}$

## Data Availability

Not applicable.
